# A Practical Finite Element Modeling Strategy to Capture Cracking and Crushing Behavior of Reinforced Concrete Structures

**DOI:** 10.3390/ma14030506

**Published:** 2021-01-21

**Authors:** Alexandre Mathern, Jincheng Yang

**Affiliations:** 1 Division of Structural Engineering, Concrete Structures, Department of Architecture and Civil Engineering, Chalmers University of Technology, SE-412 96 Gothenburg, Sweden; 2 Research and Innovation, NCC, Lilla Bomen 3c, SE-411 04 Gothenburg, Sweden; 3 Division of Structural Engineering, Steel and Timber Structures, Department of Architecture and Civil Engineering, Chalmers University of Technology, SE-412 96 Gothenburg, Sweden

**Keywords:** reinforced concrete, finite element analysis, crack band, strain localization, post-peak softening, viscoplastic regularization, convergence, mesh sensitivity, bond–slip, flexural behavior

## Abstract

Nonlinear finite element (FE) analysis of reinforced concrete (RC) structures is characterized by numerous modeling options and input parameters. To accurately model the nonlinear RC behavior involving concrete cracking in tension and crushing in compression, practitioners make different choices regarding the critical modeling issues, e.g., defining the concrete constitutive relations, assigning the bond between the concrete and the steel reinforcement, and solving problems related to convergence difficulties and mesh sensitivities. Thus, it is imperative to review the common modeling choices critically and develop a robust modeling strategy with consistency, reliability, and comparability. This paper proposes a modeling strategy and practical recommendations for the nonlinear FE analysis of RC structures based on parametric studies of critical modeling choices. The proposed modeling strategy aims at providing reliable predictions of flexural responses of RC members with a focus on concrete cracking behavior and crushing failure, which serve as the foundation for more complex modeling cases, e.g., RC beams bonded with fiber reinforced polymer (FRP) laminates. Additionally, herein, the implementation procedure for the proposed modeling strategy is comprehensively described with a focus on the critical modeling issues for RC structures. The proposed strategy is demonstrated through FE analyses of RC beams tested in four-point bending—one RC beam as reference and one beam externally bonded with a carbon-FRP (CFRP) laminate in its soffit. The simulated results agree well with experimental measurements regarding load-deformation relationship, cracking, flexural failure due to concrete crushing, and CFRP debonding initiated by intermediate cracks. The modeling strategy and recommendations presented herein are applicable to the nonlinear FE analysis of RC structures in general.

## 1. Introduction

Finite element (FE) analysis is effective for investigating the nonlinear behavior of reinforced concrete (RC) structures and performing parametric studies at lower costs than experimental tests. The nonlinear FE analysis of RC members has been extensively reported, and good agreement is often achieved between numerical and experimental results [1,2,3]. However, the comparison and application of the existing FE models are difficult owing to the differences in the adopted modeling strategies, which involve a considerable number of options, e.g., regarding the concrete constitutive models, critical parameters, bond between the concrete and the steel reinforcement, and numerical analysis procedures. Nonlinear FE analyses for blind predictions of the ultimate capacity and cracking of simple RC structural members have been associated with large uncertainty [4,5].

The major challenges in the nonlinear FE analysis of RC structures include the following: (1) defining the concrete tensile and compressive behaviors with the proper consideration of the strain localization in fracture zones [6], (2) efficiently assigning proper bond–slip behavior between the concrete and the steel reinforcement [2], (3) solving convergence difficulties commonly observed in the modeling of concrete with high nonlinearity [7,8], and (4) the misinterpretation of the processing logic of the FE software in the derivation and definition of input data. These challenges, which are described in detail below, render nonlinear FE analyses of RC complex and time-consuming; thus, such nonlinear analyses are rarely performed by practicing engineers.

*Concrete cracking in tension*: The cracking of tensile concrete is usually modeled by either discrete or smeared crack approach in practical FE analyses. In the discrete crack approach, physical cracks are modeled as displacement discontinuities in a concrete continuum. Although it allows the precise prediction of localized deformation at cracks, the discrete crack approach requires pre-defining tensile fracture zones when the finite element is generated. However, the position of the cracks is not known beforehand for most structural analyses. To overcome this limitation, automated re-meshing techniques are required to adapt the configuration of finite elements in accordance with the propagating cracks [9,10]. Although recent research has been devoted to developing discrete crack models allowing arbitrary crack initiation and propagation (e.g., the extended FE method [11]), the sophisticated modeling methods are not suitable for practical application by engineering practitioners. The ease of application motivates the wide use of the smeared crack approach in practical FE analyses. In the smeared crack approach, a crack width is transformed into an equivalent cracking strain smearing over a certain length (referred to as the “smeared length” in this paper). The numerical results correspond to reality only if the widths of the simulated fracture process zones (i.e., the cracking regions) are equal to the assumed smeared length [6,12,13]. To assure the reliability of the numerical results, the smeared length should be properly determined and incorporated into the definition of the constitutive law of concrete in tension.

*Concrete crushing in compression*: The constitutive model for concrete in uniaxial compression is usually provided as a stress–strain relationship in design codes, e.g., Model Codes [14,15] and ACI 318 [16]. However, such as a compressive strain, i.e., the “mean strain” obtained by smearing the measured deformation over the length of the standard test specimen, cannot describe the local strain-softening behavior in the critical fracture damaged zone of concrete. Studies since the 1980s [17,18,19] have investigated the effects of the strain localization on deriving the concrete compressive constitutive models. The challenge faced when considering the strain localization in FE analyses is that the actual size of simulated fracture zones is not known in advance, but it must be determined and used to modify the constitutive model of concrete as input data for the FE analyses. Zandi Hanjari et al. [20] modified the post-peak branch of the stress–strain relationship proposed by Thorenfeldt et al. [21], assuming that concrete crushing occurred in one row of concrete elements in the FE analyses of RC members. To properly define the concrete compressive behavior and reliably predict the capacity of RC members governed by concrete crushing, it is necessary to (1) clarify the principle of modifying the concrete compressive constitutive model with consideration of the strain localization and (2) develop a practical approach for determining the size of the fracture zones.

*Bond–slip behavior between concrete and steel reinforcement*: In the nonlinear FE analyses of RC structures, the definition of the bond–slip behavior between the concrete and the steel reinforcement is critical for the accurate prediction of the structural responses, crack patterns, and crack widths [2,3]. However, there is a lack of guidelines and different methods are used in the literature to assign the steel-concrete interaction, requiring different input data and workarounds to overcome implementation difficulties, which are often not described in detail [22,23]. Therefore, it is important for the research community to evaluate the existing approaches [2,24,25] for assigning the bond–slip behavior and to develop new approaches involving simple application procedures.

*Convergence difficulties*: The softening behavior and stiffness degradation of cracking or crushing concrete cause severe convergence difficulties in the static analysis of concrete [8,26]. Instead of solving the problem in static analyses, researchers implemented dynamic analysis procedures adopting implicit [7] or explicit [1] integration methods. However, such a dynamic approach requires additional effort to carefully select, e.g., the time integration algorithm, loading scheme, loading time, damping ratio, and time increment size, to achieve a good balance between minimizing the inertial forces (for a better approximation of the static problem) and reducing the computational time (by using a shorter time to model the static event in an accelerated manner). Therefore, it remains important to develop a simple solution strategy to perform the static analysis procedure with a high convergence rate.

*Misinterpretation*: Misinterpretation refers to the users’ misunderstanding of the processing logic of FE software packages. Misinterpretation may cause the incorrect definition of input data, which may lead to errors in numerical results or aborted analyses [27]. For instance, in nonlinear FE analyses of concrete structures, the definition of the post-peak softening behavior of concrete constitutive laws or the stiffness degradation of damaged concrete is not straightforward. Furthermore, such analyses require to assign a lot of input data and to make a great number of modeling choices, which are rarely reported in a very detailed way in the literature. This is explained by the fact that such details do not constitute the focus of the investigation and are usually software specific. Nevertheless, if the approach for obtaining critical input data is not reported, it can undermine the reliability and reproducibility of the FE analyses.

In light of these challenges, the objective of this study was to develop a robust and reliable modeling strategy to capture the tensile cracking and compressive crushing behavior of RC structures associated with low computational costs and ease of implementation, based on the well-established constitutive relations from fib Model Codes [14,15]. The strategy was implemented to simulate the flexural behavior of an RC beam as reference and another identical RC beam strengthened with an externally bonded carbon-reinforced polymer (CFRP) laminate; both beams were tested in four-point bending until failure. Modeling of crack opening after the reinforcement yielding stage and ultimate concrete crushing were carefully studied on the reference beam to ensure a reliable basis for the modeling of the strengthened beam. The nonlinear FE analyses presented herein were performed using the concrete damaged plasticity (CDP) model implemented in the commercial software ABAQUS [26], as it is widely used in both academia and industry to analyze RC structures [28,29,30,31,32]. The focus of this paper was to provide reliable, practical, and computationally cost-efficient implementation guidelines for nonlinear FE analyses of concrete structures, which can be used as a basis for more complex cases and support the application of nonlinear analyses to real-world engineering problems, e.g., for load-bearing assessment, strengthening assessment, structural health monitoring, and damage identification of building and civil engineering structures. For instance, the use of externally bonded FRP laminates for strengthening and rehabilitation of concrete or masonry structural members [33,34,35] has emerged as an effective technique and found strong interest in both research and practice, which supported the consideration of such a case in this work. The experimental setup is shown in Section 2. In Section 3, the modeling procedures and recommendations for overcoming the aforementioned challenges are presented in detail. In Section 4, the proposed modeling strategy is demonstrated, and modeling choices are validated by parametric studies considering the reference RC beam. In Section 5, the numerical results of the reference beam are compared with the experimental measurements regarding load-deformation relationship, cracking, flexural failure due to concrete crushing, and CFRP debonding initiated by intermediate cracks.

## 2. Experimental Test

**Figure 1:**
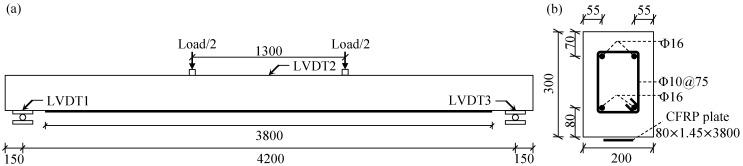
(**a**) Reinforced concrete (RC) beams subjected to four-point bending tests until failure (unit: mm); (**b**) cross-sectional dimension of the RC beams (with a carbon-reinforced polymer (CFRP) plate for the strengthened beam only).

The RC members modeled in the present FE study included two slender RC beams subjected to four-point bending in the laboratory, see Figure 1a. The RC beams were cast at a workshop using C35/45 concrete. One beam served as reference; the other one, with identical dimensions, was strengthened with an externally bonded CFRP laminate on the tensile side of the beam. The cross-sectional dimensions of the beams are shown in Figure 1b, including main steel rebars, shear reinforcement, and externally bonded CFRP plate (for the strengthened beam only). A two-component epoxy adhesive (StoPox SK41, StoCretec GmbH, Kriftel, Germany) was applied to bond the CFRP plate; whose layer design thickness was 1 mm. Mechanical properties of the above-mentioned materials used for the FE analyses are listed in Table 1: the elastic modulus $E_{c}$, compressive strength $f_{c}$, and tensile strength $f_{\mathrm{ct}}$ of concrete C35/45 at the age of 287 days were estimated according to Eurocode 2 [36]; the Poisson’s ratio of concrete $\nu_{c}$ was defined according to Model Code 2010 [15]; the elastic modulus $E_{s}$, yield strength $f_{\mathrm{sy}}$, ultimate strength $f_{\mathrm{su}}$, and ultimate strain $\varepsilon_{\mathrm{su}}$ of steel reinforcement were measured by laboratory tests on bars with a diameter of 16 mm (Φ16) according to ASTM A615 [37]; the elastic modulus $E_{f}$ and ultimate tensile strain $\varepsilon_{\mathrm{fu}}$ of the CFRP plate were measured according to standard tensile tests as reported in [38]; the elastic modulus $E_{a}$, tensile strength $f_{a},$ and Poisson’s ratio $\nu_{a}$ of the epoxy adhesive were reported in [39].

In the four-point bending tests, the beams were simply supported on two movable steel supports, giving an effective span of 4.2 m. The steel support at each end consisted of two identical steel plates (170 × 30 × 200 mm^3^) and one steel cylinder roller (diameter of 50 mm and length of 200 mm). External loading was applied via two synchronized hydraulic jacks using displacement control. The foot of each hydraulic jack rested on a steel plate (50 × 50 × 200 mm^3^) to distribute the load to the RC beam. The beams were loaded to failure. In the reference beam, flexural failure after yielding of the reinforcement was governed by concrete crushing in the compressive side of the beam; the failure of the strengthened beam was due to premature debonding of the CFRP plate initiated by intermediate flexural cracks.

During the test, strain gauges and linear variable differential transducers (LVDTs) were used to monitor the beam specimens. Two strain gauges were installed at the midspan cross-section on the tensile steel reinforcement, and three LVDTs were used to obtain the net deflection at the midspan, as shown in Figure 1. Cracks in the RC beams were monitored during the test; the crack widths were measured at the height of the tensile steel reinforcement using a digital handheld microscope (AM4115ZT, Dino-Lite Europe, Almere, The Netherlands) at load levels of 15, 30, 45, 55 (reference beam only), and 70 kN (strengthened beam only).

## 3. FE Modeling Strategy

The proposed modeling strategy is discussed in detail, in this section, with special focus on proper modeling of RC considering strain-softening in fracture zones and bond–slip between steel reinforcement and concrete to ensure the reliable prediction of cracking and crushing. The strategy is adapted to the modeling of the RC beams introduced in Section 2. However, common critical issues in the nonlinear FE modeling of RC structures are addressed in a general manner, which makes these recommendations applicable to other types of RC beam and frame structures. The nonlinear FE analyses were conducted using the commercial FE package ABAQUS/CAE, version 6.14 [26].

Considering that the beam geometry and the test configuration were symmetric about the midspan, only one half of the RC beam was modeled in the current FE analyses to reduce the computational cost. The vertical load acting on the beam was defined as a boundary condition in the FE model, inducing a vertical displacement on the top of the steel plate between the load and the beam. The interaction between the steel plate and the beam was set as surface-to-surface contact, which defined the interfacial constraints in the normal direction (i.e., “hard” contact) and friction in the tangential direction. The coefficient of tangential friction was assumed to be 0.57 according to a previous recommendation [40]. The same contact settings were used at the interfaces between the beam and steel support. On the bottom side of the movable steel support, boundary conditions were defined at the middle point to constrain the degree of freedom in the vertical direction but allow translation in the horizontal direction. The concrete beam, steel plate under the loading point, and movable steel support were modeled with 2D shell, discretized into structured meshes, and assigned with element type CPS4 (4-node plane stress quadrilateral elements with four integration points). Steel reinforcement and CFRP plate, modeled as one-dimensional (1D) wire, were assigned with truss (T2D2) and beam (B21) elements, respectively. Material properties of the concrete, steel reinforcement, and CFRP were defined according to the values in Table 1. Details about the modeling of concrete, the interaction at the concrete and steel reinforcement interface and the concrete and CFRP plate interface, and the numerical solution strategy are described below according to the proposed modeling strategy.

### 3.1. Modeling of Concrete

The concrete material is defined in the CDP model implemented in ABAQUS [26], including the definition of the concrete plasticity, the tensile behavior, the compressive behavior, and the damage evolution of the stiffness.

#### 3.1.1. Concrete Plasticity

The concrete plasticity parameters to be defined in the CDP model include (1) dilation angle φ and eccentricity factor ϵ related to the flow potential given by the Drucker–Prager hyperbolic function; (2) factors $\sigma_{b0}/\sigma_{c0}$ and $K_{c}$ related to the yield surface based on the function presented by Lubliner et al. [41] with the modifications proposed by Lee and Fenves [42] to account for the evolution of strength in tension and compression; and (3) the viscosity parameter μ to introduce viscoplastic regularization. The values of these parameters defined in the reference FE model are presented in Table 2. Default values of ϵ, $\sigma_{b0}/\sigma_{c0}$, and $K_{c}$ are assigned according to the design manual of ABAQUS [26]; the values of φ and μ are defined based on the validation discussed in Section 4.1 and Section 4.2.

#### 3.1.2. Concrete Tensile Behavior

**Figure 2:**
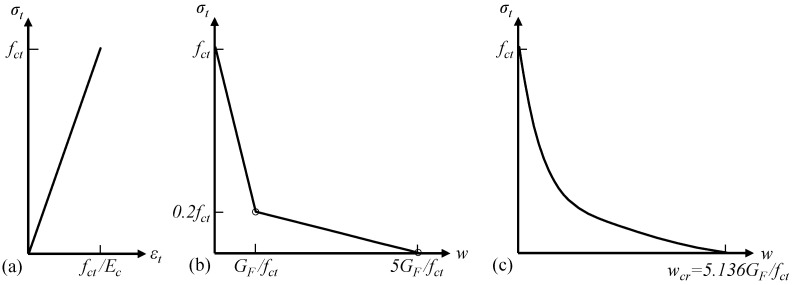
Constitutive models for concrete in tension: (**a**) linear elastic stress–strain relationship for uncracked concrete; (**b**) bilinear tensile stress–crack width relationship according to Model Code 2010 [15]; (**c**) exponential tensile stress–crack width relationship proposed by Hordijk [43].

The tensile behavior of concrete is characterized by a linear elastic stress–strain relationship before the concrete reaches the tensile strength $f_{\mathrm{ct}}$ (Figure 2a) and a bilinear stress $\sigma_{t}$-crack width w relationship for the post-peak softening behavior according to Model Code 2010 [15]. The Model Code relationship, as shown in Figure 2b, is determined by the tensile strength $f_{\mathrm{ct}}$ and the fracture energy $G_{F}$. The fracture energy $G_{F}$ describes the amount of energy required to propagate a tensile crack of unit area; for normal-strength concrete, $G_{F}$ (in N/m or J/m^2^) can be estimated using Equation (1) according to Mode Code 2010 [15]:

(1) $$G_{F}\;=\;{73f}_{c}{}^{0.18},$$

where $f_{c}$ represents the mean compressive strength of concrete in MPa.

To validate the adopted concrete tensile behavior, the effects of different modeling choices on the numerical results were investigated and are discussed in Section 4.3. This included (1) a comparison between the bilinear Model Code relation and another commonly used post-peak softening model, i.e., the exponential descending $\sigma_{t}$-w relation (Figure 2c) proposed by Hordijk [43], and (2) parametric studies of the assumed tensile strength $f_{\mathrm{ct}}$ and fracture energy $G_{F}$. The Hordijk $\sigma_{t}$-w relation is expressed by Equation (2):

(2) $$\frac{\sigma_{t}}{f_{\mathrm{ct}}}=\left[1+{\left(c_{1}\frac{w}{w_{\mathrm{cr}}}\right)}^{3}\right]e^{-c_{2}\frac{w}{w_{\mathrm{cr}}}}-\frac{w}{w_{\mathrm{cr}}}\left(1+c_{1}^{3}\right)e^{-c_{2}},$$

where, $c_{1}=3.0$, $c_{2}\;=\;6.93$, $w_{\mathrm{cr}}\;=\;5.136\frac{G_{F}}{f_{\mathrm{ct}}}$.

*Smeared crack method:* To simulate the cracks in concrete, the CDP model adopted in the present FE study employs the smeared crack method, where the cracking concrete is treated as a continuum and a physical crack opening $w_{\mathrm{cr}}$ is characterized as an equivalent cracking strain $\varepsilon_{\mathrm{cr}}$ smearing over a certain length (the smeared length $l_{s}$). Thus, the post-peak tensile behavior defined in the CDP model complies with stress–cracking strain relationship, which is converted from the selected stress–crack width model given $\varepsilon_{\mathrm{cr}}{\;=\;w}_{\mathrm{cr}}/l_{s}$. The numerical results correspond to reality only if the width of the simulated fracture process zone $l_{F}$ is equal to the assumed smeared length $l_{s}$. As indicated by Equation (3), the inelastic deformation δ(σ) of cracking concrete at a certain stress level σ is not dependent on the assumed $l_{s}$ but determined by the selected stress–crack width relation in the present FE analyses.

(3) $$\delta \left(\sigma \right)=l_{F}\varepsilon_{\mathrm{cr}}\left(\sigma \right)=l_{F}\frac{w_{\mathrm{cr}}\left(\sigma \right)}{l_{s}}=\left\{l_{F}=l_{s}\right\}=w_{\mathrm{cr}}\left(\sigma \right).$$

*Crack band approach:* To define the smeared length $l_{s}$ in accordance with the size of the simulated fracture zone $l_{F}$, the crack band approach—a simple technique for practical engineering computations—is adopted. In this approach, it is assumed that the strain-softening of cracking concrete is localized into a clear band of elements running across the concrete mesh and thus forming a “crack band.” Thus, the size of the simulated fracture zone $l_{F}$ becomes the width of the crack band $h_{b}$, and $h_{b}$ can be estimated and assigned to the smeared length $l_{s}$ to adjust the strain-softening behavior of the concrete in the post-peak regime [6]. The crack band approach, which is widely applicable and utilized in many FE packages, is based on pioneering studies performed in the 1980s [12,13,44,45]. Theoretically, the width of crack bands $h_{b}$ is a function of not only the element topology, e.g., the type, shape, size, and integration scheme, but also the crack band orientation [6]. However, the width of crack bands $h_{b}$ implemented in common FE packages, e.g., ABAQUS [26], is simply estimated as the square root of the element area (for two-dimensional elements) or the cubic root of the element volume (for three-dimensional elements). This simplified estimation may induce substantial error and mesh sensitivity for elements with large aspect ratios. It is recommended to use elements having aspect ratios close to 1 (e.g., square or cubic elements) to reduce the mesh sensitivity [26]. Even for square or cubic elements, there may be errors if the crack band is not aligned with the mesh line. For instance, for a two-dimensional mesh of square elements with side length *a*, it is reasonable to estimate the band width $h_{b}\;=\;\sqrt{A}\;=\;a$ automatically in ABAQUS only if the crack band is parallel to the element sides. If the crack band runs along the element diagonal, the appropriate width of the band is $h_{b}\;=\;\sqrt{2}a\;=\;\sqrt{2A}$ instead of $h_{b}\;=\;\sqrt{A}$. Accordingly, when using the crack band approach in FE modeling, it is recommended to define the post-peak tensile behavior of concrete by the input data of the stress–cracking strain relationship, which allows users to evaluate and determine the crack band width $h_{b}$. For a detailed discussion regarding the estimation of the crack band width with consideration of the element topology and crack band orientation, readers are referred to [12,46,47].

In the present FE analyses, the crack bands developed in the concrete mesh mainly ran parallel to the mesh lines, owing to the predominant bending effect on the beam. Thus, the crack band width $h_{b}$ was determined as the width of square-shaped concrete elements for deriving the stress–cracking strain input data.

#### 3.1.3. Concrete Compressive Behavior

For the FE analysis of RC beams in bending, the concrete compressive behavior is widely defined according to a stress–strain relationship obtained from uniaxial compression tests of standard concrete cylinders. However, the strain in the standard compressive test is the “mean” strain obtained by smearing the measured displacement over the total length of the specimen. Considering that the compressive failure of concrete is initiated by a local shear band formed in one of the fracture zones and the post-peak deformation mainly arises from such a local zone, the “mean” strain naturally underestimates the strain in the critical fracture zone. If the post-peak deformation is expressed by the mean strain, the strain-softening curve tends to depend on the geometry of the specimen [17,18]—the descent of the post-peak branch is faster for a longer specimen. For instance, the compressive stress–strain relationship provided in Model Codes [14,48] is reasonably accurate for a concrete specimen length of approximately 200 mm.

In the present FE study, the relationship based on Model Code 1990 [14] and Model Code 2010 [15] is selected as the reference constitutive model for concrete in compression. Model Code 1990 provides the part of the descending branch with strains exceeding the concrete ultimate/limit strain $\varepsilon_{c,\lim}$. To highlight the differences among existing constitutive models for concrete compressive behavior and the impacts of these differences on the predicted ultimate crushing failure of the concrete beam, two other commonly used models are also studied for comparison (see Section 4.5): the Thorenfeldt relationship and the Saenz relationship. As shown in Table 3, the Thorenfeldt relationship is based on previous studies by Tomaszewicz [49] and Thorenfeldt et al. [21], with the modifications proposed by Collins and Porasz in CEB Bulletin 193 [50]. The Saenz relationship includes modifications [51] based on a previous discussion of the compressive stress–strain equation [52].

To obtain reliable predictions of the ultimate capacity and the crushing failure of the beam in flexural loading, the post-peak strain localization should be considered in the definition of the constitutive law. The original constitutive model based on the mean strain—mainly the post-peak softening branch—must be modified to better describe the localized strain softening in the critical fracture zone with a reasonable size. The assumed size should be verified according to the size of the simulated crushing zone.

**Figure 3:**
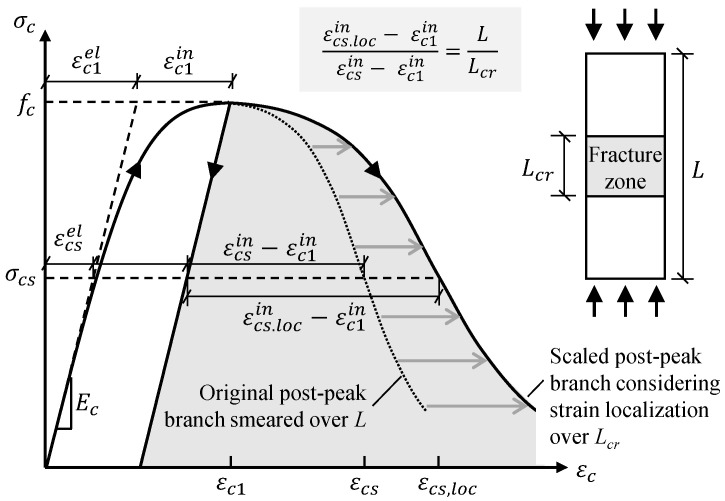
Modification of the post-peak softening branch of the constitutive model for concrete in compression originally expressed by the mean strain smearing over the whole length of specimen L to consider the strain localization in the critical fracture zone with the length of $L_{\mathrm{cr}}$.

*Modified compressive behavior considering strain localization:* The procedure for modifying the post-peak branch is presented in Figure 3. If the stress–mean strain relationship and the length *L* of the tested specimen are known, the increased post-peak deformation $\delta {|}_{c1}^{\mathrm{cs}}$ can be obtained from the stress–strain relationship using Equation (4):

(4) $$\delta {|}_{c1}^{\mathrm{cs}}=L\left(\varepsilon_{\mathrm{cs}}-\varepsilon_{c1}\right),$$

where, $\varepsilon_{c1}$ and $\varepsilon_{\mathrm{cs}}$ represent the strains (mean strains smeared over the whole specimen) corresponding to the concrete compressive strength $f_{c}$ and the stress level $\sigma_{\mathrm{cs}}$ in the softening branch, respectively.

Considering the strain localization, instead of using Equation (4), the increased deformation after the peak stress ${\delta |}_{c1}^{\mathrm{cs}}$ is reached can be calculated using Equation (5), taking into account the strain-softening in the fracture zone with the length of $L_{\mathrm{cr}}$ and the elastic unloading due to the reduction in the compressive stress from the peak stress $f_{c}$ to $\sigma_{\mathrm{cs}}$:

(5) $$\begin{array}{c} \delta {|}_{c1}^{\mathrm{cs}}=\left(L-L_{\mathrm{cr}}\right)\left(\varepsilon_{\mathrm{cs}}^{\mathrm{el}}-\varepsilon_{c1}^{\mathrm{el}}\right)+L_{\mathrm{cr}}\left[\left(\varepsilon_{\mathrm{cs}}^{\mathrm{el}}+\varepsilon_{\mathrm{cs}.\mathrm{loc}}^{\mathrm{in}}\right)-\left(\varepsilon_{c1}^{\mathrm{el}}+\varepsilon_{c1}^{\mathrm{in}}\right)\right]=L\left(\varepsilon_{\mathrm{cs}}^{\mathrm{el}}-\varepsilon_{c1}^{\mathrm{el}}\right)+\\ L_{\mathrm{cr}}\left(\varepsilon_{\mathrm{cs}.\mathrm{loc}}^{\mathrm{in}}-\varepsilon_{c1}^{\mathrm{in}}\right), \end{array}$$

where, $\varepsilon_{\mathrm{cs}}^{\mathrm{el}}-\varepsilon_{c1}^{\mathrm{el}}$ represents the change in elastic strain due to the stress reduction from $f_{c}$ to $\sigma_{\mathrm{cs}}$ in the post-peak regime, $\varepsilon_{\mathrm{cs}.\mathrm{loc}}^{\mathrm{in}}$ represents the inelastic strain localized in the critical fracture process zone, and $\varepsilon_{c1}^{\mathrm{in}}$ represents the inelastic strain at peak stress.

The transitivity between Equations (4) and (5) implies that,

(6) $$L\left(\varepsilon_{\mathrm{cs}}-\varepsilon_{c1}\right)=L\left(\varepsilon_{\mathrm{cs}}^{\mathrm{el}}-\varepsilon_{c1}^{\mathrm{el}}\right)+L_{\mathrm{cr}}\left(\varepsilon_{\mathrm{cs}.\mathrm{loc}}^{\mathrm{in}}-\varepsilon_{c1}^{\mathrm{in}}\right),$$

which can be rewritten as,

(7) $$\varepsilon_{\mathrm{cs}.\mathrm{loc}}^{\mathrm{in}}=\frac{L}{L_{\mathrm{cr}}}\left(\varepsilon_{\mathrm{cs}}^{\mathrm{in}}-\varepsilon_{c1}^{\mathrm{in}}\right)+\varepsilon_{c1}^{\mathrm{in}}.$$

The scaling rule for determining the localized inelastic strain $\varepsilon_{\mathrm{cs}.\mathrm{loc}}^{\mathrm{in}}$ in the critical fracture zone is expressed by Equation (7), as shown in Figure 3.

**Figure 4:**
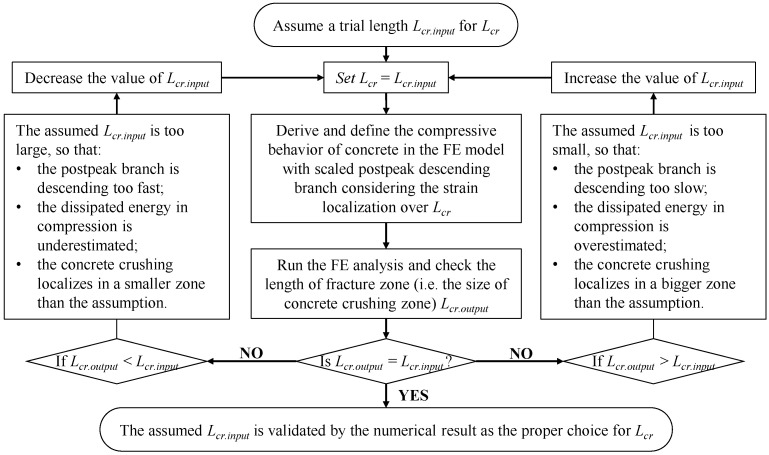
Proposed iterative procedure for identifying the reasonable length $L_{\mathrm{cr}}$ of the critical fracture zone.

*Identifying the size of crushing zone:* The modification of the post-peak strain-softening behavior also requires a proper assumption of the size of the critical fracture zone $L_{\mathrm{cr}}$, which should be validated according to the size of the simulated crushing zone in the FE analysis. Herein, an iterative procedure is proposed for identifying the value of $L_{\mathrm{cr}}$ via a reasonable approach. As indicated by the flowchart of Figure 4, the assumed value $L_{\mathrm{cr}.\mathrm{input}}$ used to modify the post-peak branch of the compressive behavior as input data should be verified according to the observed length $L_{\mathrm{cr}.\mathrm{output}}$ of the simulated crushing zone in the numerical result. $L_{\mathrm{cr}.\mathrm{output}}$ can be visualized by contour plots highlighting the regions with compressive strains larger than $\varepsilon_{c,\lim}$.

As the reference constitutive model adopted in the present FE analysis, the Model Code relation is reasonably accurate for specimens with a length of approximately 200 mm [14], providing a fair benchmark to modify the post-peak branch with the specified length L = 200 mm according to the scaling rule in Figure 3. The reasonable length of the critical fracture zone is identified as $L_{\mathrm{cr}}\;=\;100\;\mathrm{mm}$ via the proposed iterative procedure shown in Figure 4. Details regarding the identification of $L_{\mathrm{cr}}$ are presented in Section 4.5.

#### 3.1.4. Concrete Damage Evolution

**Figure 5:**
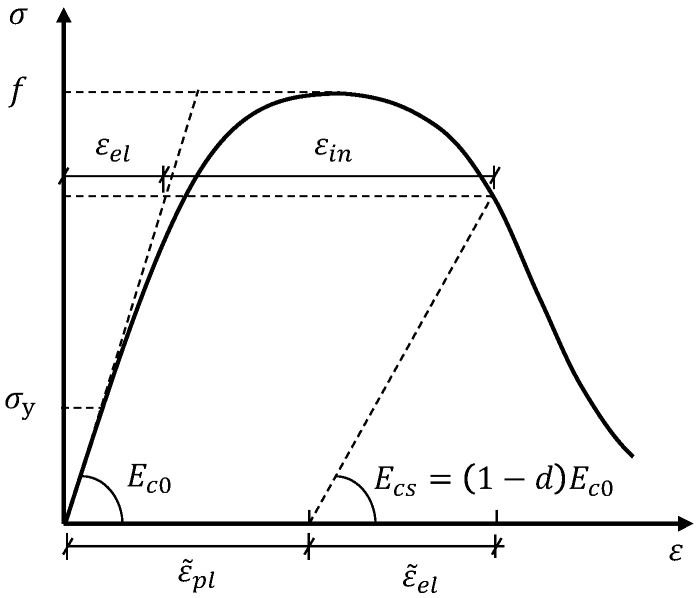
Damage evolution and degradation of the material stiffness beyond the peak stress of concrete.

In the adopted CDP model, the concrete damage evolution is characterized by the degradation of the material stiffness (i.e., the elastic modulus of concrete) in the post-peak regime of the constitutive law. Figure 5 presents a generic stress–strain relationship (Equation (8)) for concrete in uniaxial tension or compression:

(8) $$\varepsilon \left(\sigma \right)=\varepsilon_{\mathrm{el}}\left(\sigma \right)+\varepsilon_{\mathrm{in}}\left(\sigma \right)=\frac{\sigma }{E_{c0}}+\varepsilon_{\mathrm{in}}\left(\sigma \right),$$

where $\varepsilon_{\mathrm{el}}\left(\sigma \right)$, $\varepsilon_{\mathrm{in}}\left(\sigma \right)$, and ε(σ) represents the elastic, inelastic, and total strains, respectively, at a given stress σ; the inelastic strain is $\varepsilon_{\mathrm{in}}(\sigma )\;=\;0$ when ${\sigma \;<\;\sigma }_{y}$ (the inelastic strain $\varepsilon_{\mathrm{in}}$ is commonly referred to as the cracking strain $\varepsilon_{\mathrm{cr}}$ in the description of concrete in tension). $E_{c0}$ represents the initial elastic modulus of undamaged concrete.

Considering the damage evolution beyond the peak stress, the initial elastic strain $\varepsilon_{\mathrm{el}}\left(\sigma \right)$ in Figure 5 changes to ${\tilde{\varepsilon }}_{\mathrm{el}}\left(\sigma \right)$ owing to the degradation of the elastic modulus from $E_{c0}$ to $E_{\mathrm{cs}}$ according to Equation (9).

(9) $$E_{\mathrm{cs}}=E_{c0}\left(1-d\right),$$

where, d is the damage factor to be defined for characterizing the damage evolution.

In the present FE analyses, the damage model proposed by Lubliner et al. [41] is adopted, which assumes that in the post-peak regime, the degraded material stiffness is proportional to the residual cohesion of the material. Considering that the material cohesion can be correlated to the stress state, this leads to:

(10) $$\frac{E_{\mathrm{cs}}}{E_{c0}}=\frac{c}{c_{\max}}=\frac{\sigma }{f},$$

where c and $c_{\max}$ represent the material cohesion in the yield criteria corresponding to the stress level σ and peak stress f, respectively. For concrete, f represents the concrete tensile strength $f_{\mathrm{ct}}$ or compressive strength $f_{c}$.

Substituting Equation (10) into Equation (9) yields the damage variable, as follows:

(11) $$d=1-\frac{\sigma }{f}.$$

Importantly, the defined damage variable must satisfy the condition that the equivalent plastic strain ${\tilde{\varepsilon }}_{\mathrm{pl}}$ should not decrease as the damage variable increases.

### 3.2. Modeling of Steel Reinforcement

**Figure 6:**
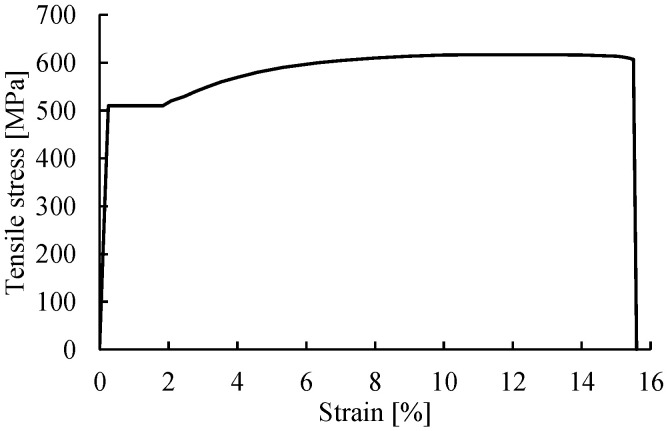
Tensile behavior defined for the steel reinforcement.

The steel reinforcement, including the longitudinal reinforcing bars and the transverse stirrups, is modeled as a one-dimensional wire, to which the element type *truss* is assigned. The material properties of the steel reinforcement are defined according to the results of standard tensile tests performed in the laboratory. Figure 6 shows the tensile stress–strain relationship defined for the steel reinforcement in the FE analyses, which is characterized by the elastic modulus $E_{s}\;=\;201\;\mathrm{GPa}$, the yielding stress $f_{\mathrm{sy}}\;=\;510\;\mathrm{MPa}$, the ultimate tensile strength $f_{\mathrm{su}}\;=\;617\;\mathrm{MPa}$, the ultimate strain $\varepsilon_{\mathrm{su}}\;=\;12.0\%$, and the rupture strain $\varepsilon_{\mathrm{smax}}\;=\;15.6\%$.

### 3.3. Interaction between Concrete and Steel Reinforcement

**Figure 7:**
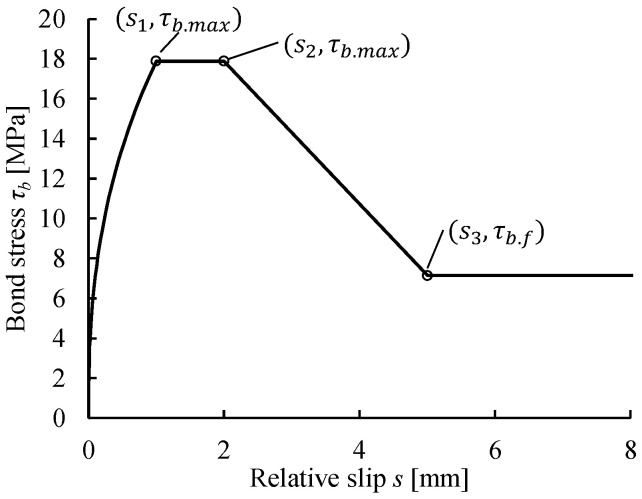
Bond–slip relation between the (longitudinal) steel reinforcement and the concrete in good bond condition according to Model Code 2010 [15].

The proper modeling of the bond–slip behavior between the concrete and the embedded steel reinforcement is critical for obtaining clear discrete crack bands in the simulated beam model and reasonable flexural responses after the cracking point. In the present FE analyses, the bond between the longitudinal reinforcement and the concrete was assumed to be in good condition, and the bond–slip relationship according to Model Code 2010 [15] is adopted. The Model Code bond–slip relationship is shown in Figure 7, where $\tau_{b.\max}\;=\;17.9\;\mathrm{MPa}$, $\tau_{b.f}\;=\;0.4\tau_{b.\max}$, $s_{1}\;=\;1.0\;\mathrm{mm}$, $s_{2}\;=\;2.0\;\mathrm{mm}$, and $s_{3}\;=\;5.0\;\mathrm{mm}$. The interaction between the stirrups and the concrete was modeled as embedded, assuming a perfect bond with no relative slip (a simplification with negligible effects).

**Figure 8:**
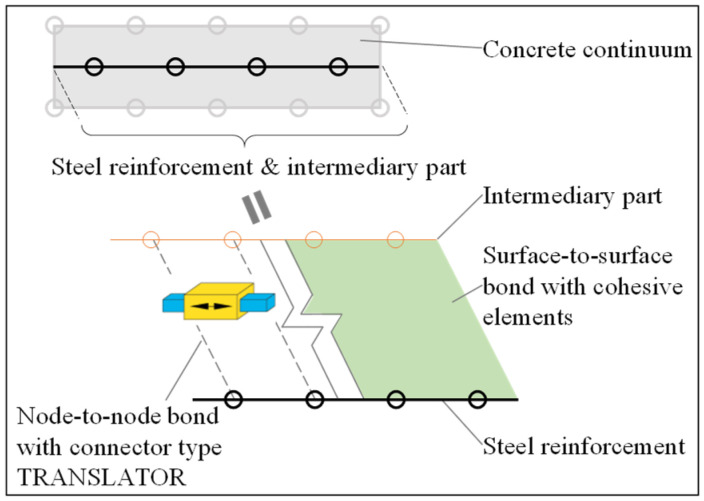
Illustration of the bond between the steel reinforcement and the concrete built using the connector method or cohesive method.

To implement the bond–slip behavior in the FE model, the use of connectors to build the node-to-node bond is proposed as a reference method. Another commonly applied method using cohesive elements [2,54] is implemented for comparison. These two methods are referred to as the connector method and the cohesive method, and corresponding schemes are presented in Figure 8. An intermediary part is created as a copy of the steel reinforcement wire but with a significantly lower material stiffness (e.g., 0.1% of $E_{s}$). The intermediary part is embedded (i.e., nodes are fully constrained) in the concrete continuum at the position of the steel reinforcement. The real steel reinforcement wire is connected to the intermediary part instead of the concrete continuum, using either connectors or cohesive elements. The chosen bond–slip relationship is finally assigned to the connectors or cohesive elements. The main benefit of introducing such an additional intermediary part is that the interaction properties assigned to the connectors or cohesive elements are not affected by the mesh refinement of the concrete continuum.

#### 3.3.1. Node-to-Node Connector Method

In the connector method, the bond–slip interaction is created using connectors (type: translator) between the nodes of the steel reinforcement and the nodes of the concrete. This method is adopted in the FE analyses as the reference modeling choice to assign the bond–slip behavior. To facilitate the assignment of node-to-node connectors, a Python script is developed to automatically implement multiple wire features between the neighboring nodes of the reinforcement and concrete. These wires can be efficiently defined using a suitable type of connector called translator, which allows uniaxial translation only in the direction of the steel reinforcement between the connected nodes. Considering that the bond–slip behavior is realized by discrete node-to-node connections, the bond force $V_{b}$, rather than the bond stress $\tau_{b}$, should be derived to define the bond at a given relative slip *s* between the steel reinforcement and the concrete, as indicated by Equation (12). When the connector method is used, for obtaining an accurate simulation of the nodal bond forces and crack widths, the connector spacing (and the length of the steel reinforcement elements) should not exceed the size of the concrete elements.

(12) $$V_{b}=\tau_{b}C_{s}s_{\mathrm{conn}},$$

where, $C_{s}$ represents the total circumference of the steel reinforcement, and $s_{\mathrm{conn}}$ represents the distance between two neighboring node-to-node connectors.

#### 3.3.2. Surface-to-Surface Cohesive Method

The cohesive method in ABAQUS can model the interfacial bond behavior in either cohesive-contact or cohesive-element approach. The cohesive-contact approach defines the cohesive behavior as part of a contact model with zero interface thickness. For instance, this approach is used in [24,25] to efficiently define multiple interfacial responses in Mode I and Mode II. As an alternative, the cohesive-element approach utilizes cohesive elements to model the bond interface with a finite thickness; thus the interfacial response is characterized by the constitutive behavior assigned to the cohesive elements [2]. The cohesive-element approach allows to easily track damage evolution in the interface and visualize the bond failure by removing the damaged cohesive elements. This is critical to capture the debonding process of the CFRP plate in the current study (see Section 3.4). Therefore, the cohesive-element approach is also used here for modeling the steel-concrete interaction in order to compare it with the proposed node-to-node connector method. The layer of cohesive elements has a negligible thickness (1 µm in the current FE models) between the steel reinforcement wire and the concrete continuum. The constitutive response of cohesive elements was defined to represent the bond stress–relative slip relationship in Figure 7; damage evolution was introduced to characterize the nonlinear response of softening and degradation of elasticity.

### 3.4. Interaction between CFRP Plate and Concrete

**Figure 9:**
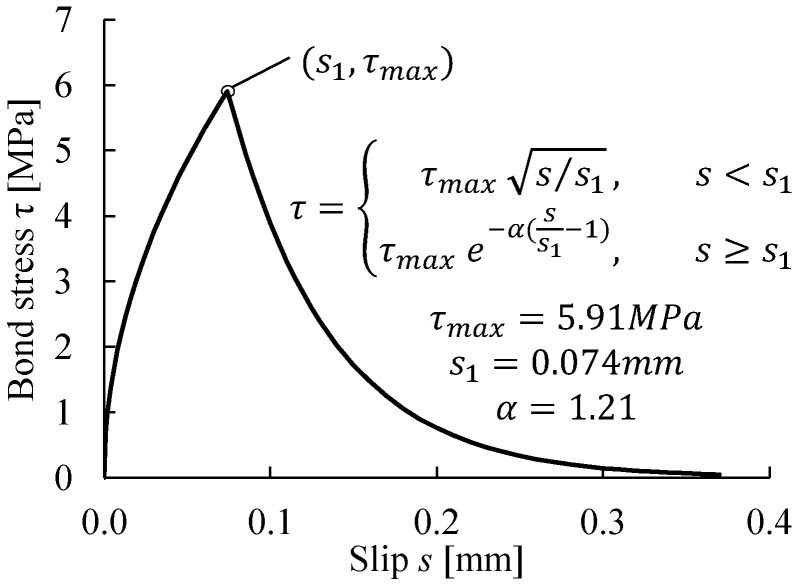
Bond–slip model assigned between the CFRP plate and concrete.

In the modeling of the CFRP-strengthened beam, the bond between the CFRP plate and the concrete was modeled in the cohesive-element approach, as described in Section 3.3.2, in order to capture the debonding process induced by intermediate cracks. The 1 mm-thick adhesive layer was modeled with cohesive elements. The constitutive response of the cohesive elements was defined to represent the bond–slip model proposed by Lu et al. [55], see Figure 9.

### 3.5. Numerical Solution Strategy

The static analysis procedure is used for the reference FE model to solve the nonlinear response of the RC beam subjected to monotonic loading. To overcome severe convergence difficulties in the static analysis [8,26], the technique of viscoplastic regularization is implemented and recommended (see Section 4.2). As an alternative to the static method, numerical analyses can be performed in a dynamic procedure adopting an implicit or explicit solution method. In Section 4.7, the dynamic analysis approach based on the implicit Hilber–Hughes–Taylor–α solution method proposed by Chen et al. (2015) [7] is implemented in comparison with the static analysis solution for validation.

## 4. Validation of Modeling Choices and Parametric Studies

According to the proposed modeling strategy, the modeling choices adopted in the reference FE model were validated and analyzed via multiple groups of parametric studies. Additionally, through the parametric studies, the effects of available modeling alternatives were investigated, and the impacts of essential parameters were quantified.

### 4.1. Dilation Angles

The dilation angle—one of the parameters defining the concrete plasticity—should be positive and in the range of 0°–56.3°. Malm (2009) [8] performed a parametric study of the dilation angle in FE analyses of an RC beam subjected to a four-point bending test. The results indicated that a dilation angle between 30° and 40° yielded converging load–deflection curves having a good agreement with the experimental flexural behavior. Jankowiak and Lodygowski (2005) [56] performed flexural tests on RC concrete beams in a laboratory to identify the reasonable value of the dilation angle required in the CDP model. A dilation angle of φ = 38° (and ϵ = 1.0) resulted in the best fit between the simulated flow potential surface and the experimental results of concrete beams ($f_{\mathrm{ct}}\;=\;2.8\;\mathrm{MPa}$ and $f_{c}\;=\;50\;\mathrm{MPa}$). Therefore, in the proposed reference FE model, the dilation angle φ was set as 35°. This value was also used in previous studies of RC beams [2,8,57].

**Figure 10:**
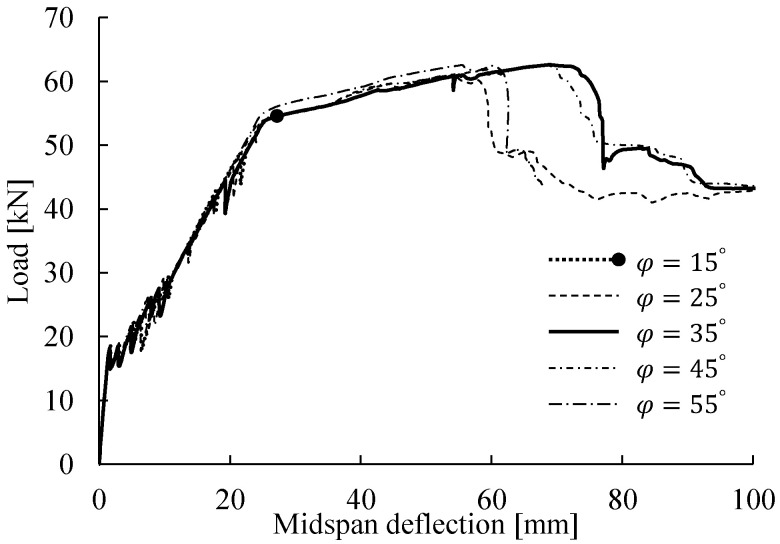
Load–midspan deflection relations for different dilation angles ranging from 15° to 55°.

Additionally, a parametric study was performed in the present study to investigate the effects of the dilation angle on the numerical results. Figure 10 shows the bending responses for different dilation angles. The FE results indicated that φ = 35° was a suitable choice providing fast convergence and a reasonable response comparable to the experimental result. 35° ≤ φ ≤ 45° appeared to be a reasonable range, as the predicted load–deflection curves converged to a similar flexural response and closely matched the experimental response. However, a value lower (φ = 25°) or higher (φ = 55°) than this range likely caused underestimation of the concrete resistance to crushing failure, leading to failure (maximum load) at a smaller deflection. According to this parametric study and the results of previous studies [2,8,57], the dilation angle of φ = 35° was validated and recommended for the modeling of similar slender concrete beams subjected to bending.

### 4.2. Viscosity Parameter

As mentioned in Section 3.1.1, the viscosity parameter can be prescribed for viscoplastic regularization in the FE analysis to overcome the severe convergence difficulties in nonlinear concrete problems using the static analysis procedure [26,58]. The viscoplasticity introduced by μ permits stresses to be outside of the yield surface, improving the convergence rate for the damaged concrete in the strain-softening regime. The default setting is μ = 0, indicating that no viscoplastic regularization is introduced.

**Figure 11:**
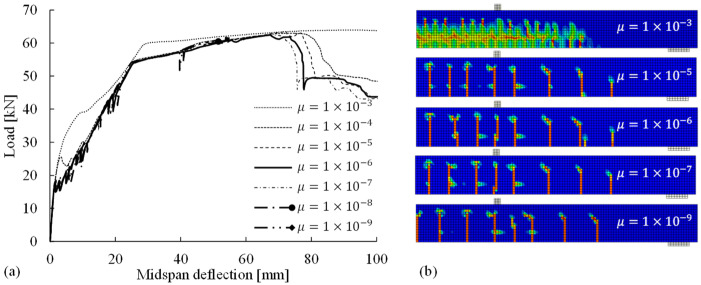
Numerical results: (**a**) load–midspan deflection relations; (**b**) crack patterns in the RC beam given the viscosity parameter *µ* ranging from $10^{-3}$ to $10^{-9}$.

To appropriately introduce the viscoplastic regularization without compromising the results, it is important to define an appropriate value of the viscosity parameter *µ*, which is theoretically smaller than the characteristic increment in the step of the nonlinear solution [26]. According to the checking of the automatically divided size of increments in the loading step, a parametric study of the viscosity parameter was performed (in the range of $10^{-3}$–$10^{-9}$ with a tenfold decrease) to identify the reasonable value for the reference FE model. The simulated load–deflection curves and crack patterns are shown in Figure 11, indicating the following: (1) a *µ* value that is too large (e.g., ${\mu \;=\;10}^{-3}{\;\mathrm{or}\;10}^{-4}$) tends to reduce the accuracy of the numerical results, leading to overestimation of the bending response of the beam and the inability to obtain clear crack patterns with localized crack bands; (2) a *µ* value that is too small (e.g., $\mu \;=\;10^{-8}\;\mathrm{or}\;10^{-9}$), although not affecting the simulated flexural behavior, can reduce the convergence rate and even abort the analysis owing to convergence difficulties; and (3) the suitable *µ* range appears to be $10^{-5}$–$10^{-7}$. Within such a range, the FE analyses not only effectively overcome the convergence problems arising from concrete cracking and crushing but also yield converging results with negligible differences regarding the load–deflection curves and the crack patterns.

According to the parametric study, the value of the viscosity parameter was selected as $10^{-6}$ in the reference FE model. The use of the viscoplastic regularization technique to solve the convergence problems in the static analysis procedure is highly recommended. However, the viscosity parameter for a specific model should be carefully selected according to parametric studies.

### 4.3. Concrete Tensile Behavior

**Figure 12:**
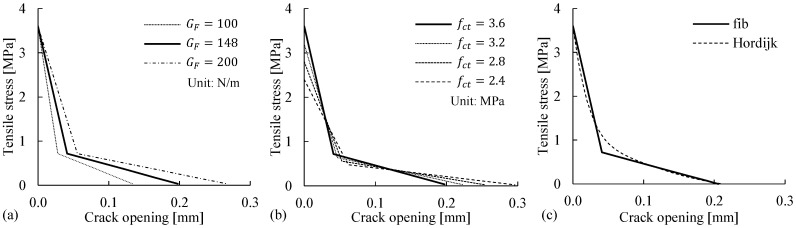
Concrete tensile behaviors considering (**a**) the uncertainty of the fracture energy $G_{F}$, (**b**) pre-cracking at a lower tensile strength $f_{\mathrm{ct}}$, and (**c**) the differences among post-peak softening models, e.g., between Model Code 2010 [15] and Hordijk [43].

**Figure 13:**
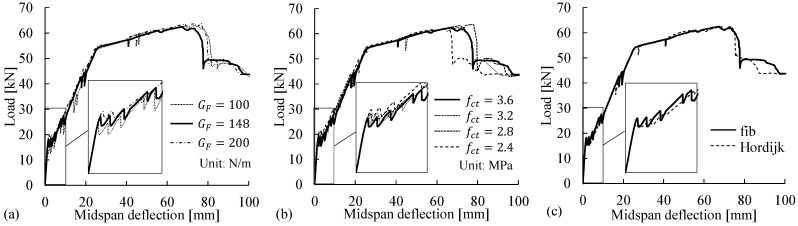
Numerical results for the load–midspan deflection relations obtained in the parametric studies on the modeling choices for (**a**) the fracture energy $G_{F}$, (**b**) the tensile strength $f_{\mathrm{ct}}$, and (**c**) the post-peak softening behavior.

The concrete tensile behavior is mainly characterized by the concrete tensile strength $f_{\mathrm{ct}}$, the fracture energy $G_{F}$, and the post-peak behavior (i.e., the shape of strain-softening curve after $f_{\mathrm{ct}}$ is reached). Considering the uncertainty of the parameters (i.e., $f_{\mathrm{ct}}$ and $G_{F}$) and the differences among the available post-peak constitutive models (see Figure 12), the modeling choices adopted for the reference FE model were validated through parametric studies of their impacts on the flexural responses (Figure 13) and cracking patterns.

Although the fracture energy $G_{F}$ in the reference model was defined as 148 N/m according to Equation (1), the parametric study covered the wide range of 100–200 N/m for the concrete material with a strength of $f_{c}\;=\;51.1\;\mathrm{MPa}$, according to fib Bulletin 42 [48]. The concrete tensile strength $f_{\mathrm{ct}}\;=\;3.6\;\mathrm{MPa}$ was defined in the reference model, and lower tensile strengths were assumed in the parametric study to investigate their effects, considering that cracks were observed before the testing date. In the parametric study of the post-peak softening behaviors, the commonly used stress–crack opening model proposed by Hordijk [43] was implemented and compared with the bilinear softening model adopted in the reference FE model according to Model Code 2010 [15]. The numerical results for the load–deflection curves (Figure 13) and crack patterns were reviewed. The results indicated that in general, the modeling choices for the three variables had negligible effects on the simulated load–deflection curves and cracking patterns, with the following exceptions: (1) there was a small difference in the first load drop after the cracking point, and (2) the number of cracks decreased from 8 to 7 when $f_{\mathrm{ct}}$ was reduced to 2.4 MPa.

### 4.4. Mesh Sensitivity Analysis

**Figure 14:**
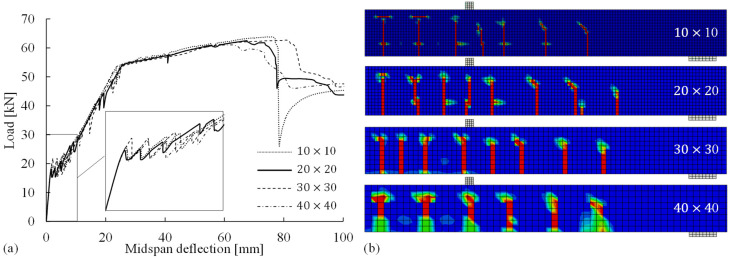
(**a**) Load–midspan deflection relations and (**b**) crack patterns for concrete element sizes of 10, 20, 30, and 40 mm.

As discussed in Section 3.1.2, the crack band approach was implemented to address the sensitivity of the numerical results to the concrete element sizes when the smeared crack method was applied to model cracks in the concrete continuum. Therefore, it was necessary to perform a mesh sensitivity analysis to verify the size-independence of the simulated results. As shown in Figure 14, the concrete continuum of each model was discretized into square elements with sizes (i.e., side lengths) ranging from 10 to 40 mm. Clearly, the simulated load–deflection curves were not sensitive to the mesh discretization. With regard to the accuracy and computational efficiency, the element size of 20 mm (20 × 20 in Figure 14) appeared to be suitable for the reference FE model, as (1) the element size of 10 mm required a longer computational time and (2) mesh sizes of >30 mm (e.g., 40 × 40 in Figure 14) resulted in a smaller number of cracks and led to the underestimation of the resistance to crushing failure in the concrete compressive zone, as the height of the compressive zone after the yielding of the steel reinforcement was less than 40 mm.

### 4.5. Strain-Softening Behavior of Concrete in Compression

In the modeling of the concrete compressive behavior, it is important to (1) consider the strain localization in the critical fracture zone and (2) accordingly modify the post-peak strain-softening behavior of the concrete in compression with the verified size of the crushing zone $L_{\mathrm{cr}}$.

**Figure 15:**
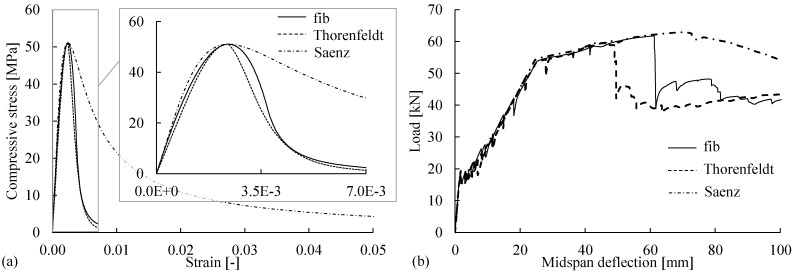
(**a**) Stress–strain models—Model Code [14,15], Thorenfeldt [21], and Saenz [51]—for the concrete compressive behavior with different post-peak softening branches; (**b**) load–deflection curves for FE analyses based on the stress–strain models without modification.

As mentioned in Section 3.1.3, the stress–strain models characterized by the mean strain tend to be size-dependent in the post-peak regime and cannot account for the strain localization in the fracture zone. For instance, the commonly used stress–strain relationships in Table 3 have different post-peak softening branches, as shown in Figure 15a. The differences in the softening behaviors significantly affect the simulated flexural responses, particularly in the ultimate state, as shown in Figure 15b. Thus, properly modifying the softening behavior is necessary for accurately simulating the flexural failure of beams governed by concrete crushing.

**Figure 16:**
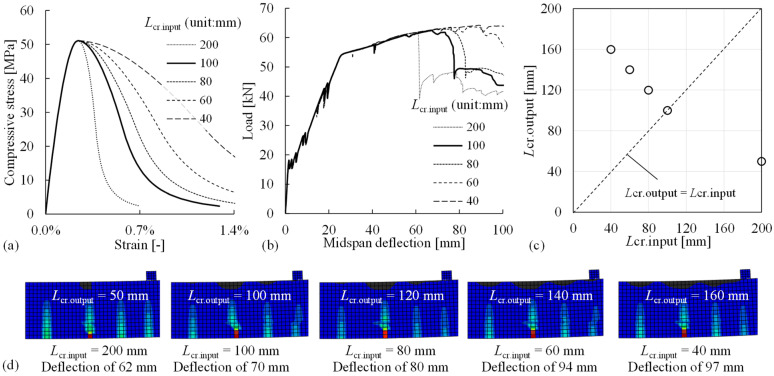
(**a**) Concrete compressive behavior based on the Model Code relation with the modified post-peak strain-softening branches given different $L_{\mathrm{cr}.\mathrm{input}}$ values ranging from 40–200 mm; (**b**) simulated load–deflection relations corresponding to each value of $L_{\mathrm{cr}.\mathrm{input}}$; (**c**) comparison of the assumed $L_{\mathrm{cr}.\mathrm{input}}$ and the observed size of the critical crushing zone $L_{\mathrm{cr}.\mathrm{output}}$ for each FE analysis (represented by a circle); (**d**) contour plot of the beam models within the constant-moment region for visualizing the crushing zones and evaluating $L_{\mathrm{cr}.\mathrm{output}}$ given different values of $L_{\mathrm{cr}.\mathrm{input}}$.

The iterative procedure proposed in Figure 4 was followed to determine the actual length of the critical crushing zone $L_{\mathrm{cr}}$ for the reference FE model. Figure 16a presents the Model Code relation, including the original post-peak branch ($L_{\mathrm{cr}.\mathrm{input}}\;=\;200\;\mathrm{mm}$) and modified post-peak branches given an $L_{\mathrm{cr}.\mathrm{input}}$ of 40–100 mm. Given the iterative assumption of $L_{\mathrm{cr}.\mathrm{input}}$ to modify the compressive behavior, the simulated flexural responses are shown in Figure 16b. The contour plot in Figure 16d shows the field output of strains within the constant-moment region of the beam model, where the concrete crushing zones are represented by the black areas with compressive-strain values $>\varepsilon_{c,\lim}$. The lengths of the simulated crushing zones $L_{\mathrm{cr}.\mathrm{output}}$ (in the compressive direction) were evaluated and compared with the assumed length $L_{\mathrm{cr}.\mathrm{input}}$ in each analysis, as shown in Figure 16c. When the size of the critical crushing zone was assumed to be 100 mm ($L_{\mathrm{cr}.\mathrm{input}}\;=\;100\;\mathrm{mm}$), the assumption was verified by the simulated crushing zone ($L_{\mathrm{cr}.\mathrm{output}}\;=\;100\;\mathrm{mm}$). Therefore, in the reference FE model, the actual length of the critical fracture zone (i.e., compressive crushing zone) was selected as $L_{\mathrm{cr}}\;=\;100\;\mathrm{mm}$, and this value was used to modify the post-peak strain-softening behavior of the concrete in compression.

### 4.6. Bond between Steel Reinforcement and Concrete

The modeling choices to be validated for the bond between the steel reinforcement and the surrounding concrete include the method of assigning the bond and the bond–slip relationship.

**Figure 17:**
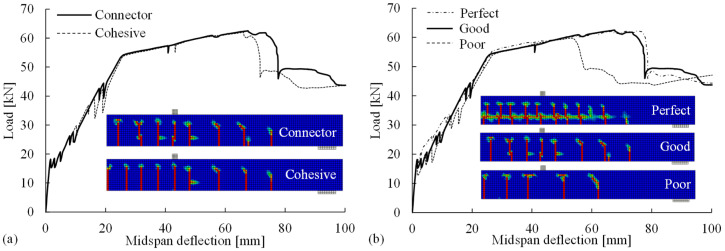
Comparisons of (**a**) methods for assigning the steel-concrete bond and (**b**) bond–slip behaviors based on the numerical results for the load–deflection relations and crack patterns.

The proposed connector method for assigning the steel-concrete bond was implemented in the reference model and compared with the cohesive-element method for validation. The numerical results for the flexural responses and crack patterns are shown in Figure 17a, indicating that the proposed connector method can provide comparable results to the cohesive method. No obvious difference was observed in the computational time between these two bond methods.

The effects of the bond–slip behaviors (perfect, good, and poor bonds) on the flexural behavior and crack pattern in the FE analyses were investigated. In a perfect bond, all the degrees of freedom of the reinforcement nodes were constrained to the nearby concrete nodes, and there was no relative slip. The good and poor bond behaviors refer to the two bond–slip relationships provided in Model Code 2010 [15] corresponding to “good” and “other” bond condition, respectively. As shown in Figure 17b, a comparison of the flexural responses revealed that a weaker bond (poor bond) caused the earlier occurrence of flexural failure due to concrete crushing, whereas the perfect bond led to a stronger response after the cracking point. The significant differences in the crack patterns indicate that selecting a suitable bond stress–slip relation is critical for the accurate simulation of cracks, e.g., a poor bond relation led to the underestimation of the number of cracks. Thus, the Model Code relationship for good condition (good bond) is a better option than the one for poor bond in order to deliver flexural responses and crack patterns comparable to the experimental observations.

### 4.7. Static and Dynamic Analysis Procedures

**Figure 18:**
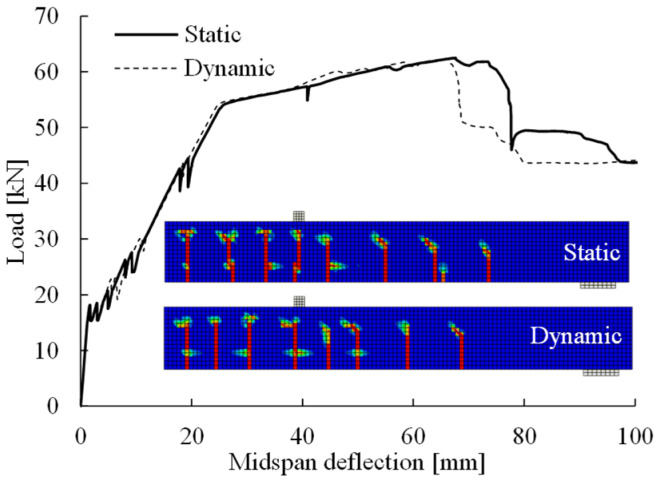
Comparison of the solution strategies between the static general analysis and the dynamic implicit analysis for the load–deflection relations and crack patterns.

The static analysis procedure (aided by the viscoplastic regularization technique) was defined in the reference FE model. It was compared with an FE model using dynamic implicit analysis based on the approach proposed by Chen et al. [7] for validation. In the dynamic analysis, critical choices and parameters included (1) ramp loading scheme, (2) load time $t_{0}{\;=\;180T}_{1}$, where $T_{1}\;=\;0.033$ s is the period of the fundamental vibration mode of the beam found from an eigenvalue analysis of the FE model period, (3) viscous damping ratio ξ = 0.05, and (4) time increment size as $T_{1}/100$. The corresponding numerical results are presented in Figure 18, which shows well-matched flexural responses until the maximum load of 62 kN with a deflection of 67 mm. The two methods predicted the same number of cracks and similar crack distributions, although there were minor differences in the positions of the cracks formed in the shear region. Thus, the static analysis procedure adopted in the reference FE model was validated, as the predicted flexural response and crack pattern were similar to those obtained via the dynamic approach.

## 5. Comparison of FE Predictions with Experimental Results

The FE models of the reference RC beam and the CFRP-strengthened beam followed the modeling strategy introduced in Section 3 and adopted the reference modeling choices for RC validated in Section 4. The numerical results of the reference beam were compared with the experimental measurements to examine the reliability of the predicted flexural response, crack pattern, crack widths, and ultimate failure due to concrete crushing. The accurate simulation of the reference beam laid the foundation for simulating the critical failure of CFRP debonding initiated by intermediate flexural cracks. The FE analysis of the CFRP-strengthened beam was chosen to show the applicability of the proposed modeling strategy for RC members with additional complexity.

### 5.1. Reference RC Beam

**Figure 19:**
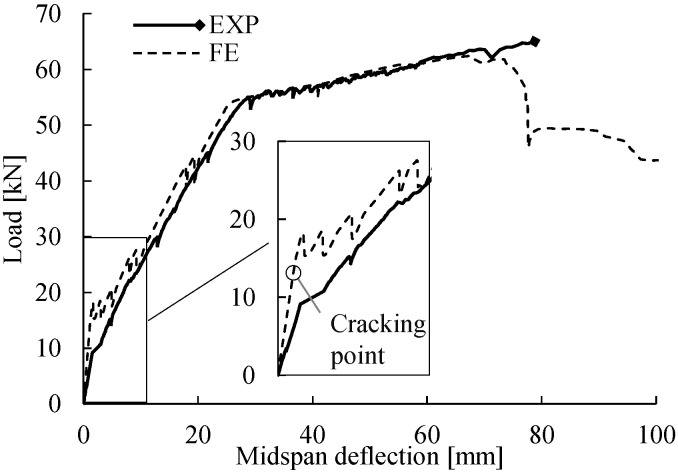
Load–deflection curves of the reference RC beam from experimental measurements (EXP) and FE analysis (FE).

The flexural responses are expressed as load–deflection behaviors in Figure 19. In general, the load–deflection curve obtained from FE analysis matched the experimental measurements; however, there were small differences in the cracking point and the ultimate failure governed by concrete crushing. The weaker response measured in the cracking stage of the experimental test was attributed to minor cracks present in the RC beam before the test because of the concrete shrinkage and unexpected loading during the transportation from the workshop to the laboratory. In the ultimate state, the FE analysis predicted that the flexural failure would be initiated at a maximum load of 62.5 kN (midspan deflection of 68 mm), whereas the loading process in the experimental test stopped (at 65.0 kN) when concrete crushing was observed at the deflection of 79 mm. Although the FE analysis slightly underestimated the ultimate capacity of the flexural failure, the degradation of the flexural response was simulated well without convergence problems.

**Figure 20:**
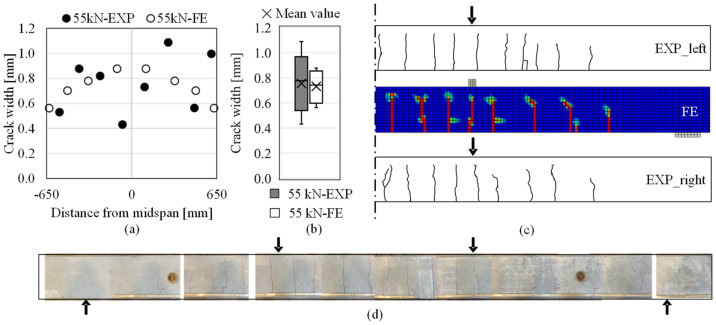
Comparison of cracks formed in the reference RC beam at the load of 55 kN regarding (**a**) crack pattern; (**b**) crack widths in the constant moment region (EXP: experimental results, FE: numerical results); (**c**) crack widths compared in box-and-whisker plots and (**d**) assembled photos depicting the crack pattern after the flexural test.

*Crack patterns and crack widths:* The crack pattern documented at the load of 55 kN (the last measurement round) is shown in Figure 20c. Although the FE analysis predicted one less crack in half of the constant-moment region, the simulated crack pattern, in general, was comparable to the distribution of cracks in the experimental test.

The experimental and numerical results of crack widths within the constant moment region are presented in Figure 20a and compared in box-and-whisker plots in Figure 20b depicting the quartiles, the variability, the median and mean values of the datasets. The boxplot of the crack widths at the load of 55 kN indicated that the mean and median values were similar between the experimental and numerical results, although there was larger variability in the experimentally measured crack widths. After reaching 55 kN, no new cracks occurred until the end of loading period; Figure 20d depicts the crack pattern on the beam after the flexural test.

### 5.2. CFRP-Strengthened RC Beam

Reliable prediction of the fast-growing crack openings after the yield point is crucial, as it is the foundation of advanced modeling with a focus on the consequential issues triggered by the opening of cracks. This was demonstrated by the modeling of the CFRP-strengthened beam; numerical results of the strengthened beam were compared to the experimental measurements with respect to its flexural response, concrete cracking, and CFRP debonding.

**Figure 21:**
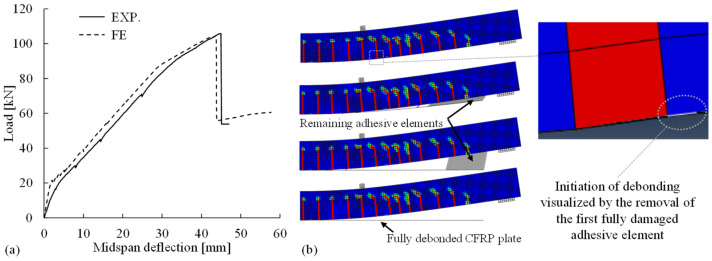
(**a**) Comparison of the flexural responses between experimental measurements (EXP) and numerical results (FE) and (**b**) intermediate cracks (IC) debonding of CFRP plate simulated in the FE analysis.

*Flexural behavior and CFRP debonding:* The flexural responses of the CFRP-strengthened beam are depicted with load–deflection curves in Figure 21a. To be highlighted, the FE analysis predicted the debonding of CFRP plate induced by intermediate cracks (IC debonding) at a maximum load of 104 kN (deflection 44 mm), which matched well with the experimental observation of IC debonding at 105 kN (deflection 45 mm). The development of IC debonding captured in the numerical simulation is shown in Figure 21b, including the initiation at the intermediate flexural crack and the evolution visualized by the removal of fully damaged adhesive (modeled as cohesive elements).

**Figure 22:**
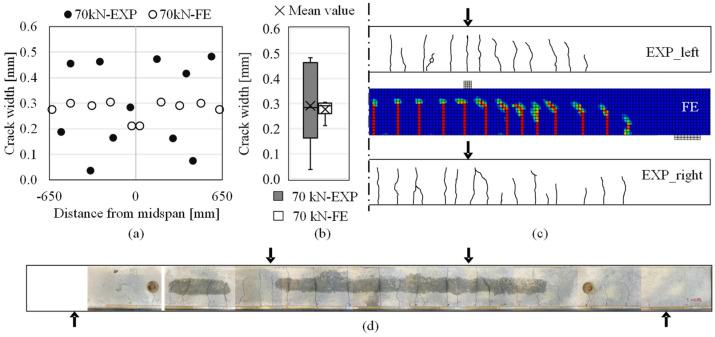
Comparison of cracks formed in the CFRP-strengthened RC beam at the load of 70 kN regarding (**a**) crack pattern; (**b**) crack widths in the constant moment region (EXP: experimental results, FE: numerical results); (**c**) crack widths compared in box-and-whisker plots and (**d**) assembled photos depicting the crack pattern after the flexural test.

*Crack patterns and cracks widths:* Reliable prediction of the IC debonding is highly dependent on the accurate simulation of cracks in the RC beam based on proper modeling options. Crack pattern and crack widths in the FE analysis were checked with the experimental measurements at different load levels. For example, Figure 22a–c demonstrates the cracks experimentally measured at the load of 70 kN (the last measurement round) in comparison to the numerical results at the same load. After 70 kN, no new cracks occurred until the end of loading period; Figure 22d depicts the crack pattern on the beam after the flexural test.

As shown in Figure 22c, localized crack bands were formed and distributed in a clear pattern, which was comparable to the experimental observation. Crack openings in the constant moment region are shown in Figure 22a–b; the crack widths less than 0.1 mm are corresponding to the measurement of two minor cracks on each side of the beam. Although the crack widths measured in the experiment showed larger variability than the simulated ones, both the median and the mean value of the experimental measurements were well captured in the numerical simulation. The reliable simulation of crack openings laid the foundation for capturing the IC debonding of CFRP in Figure 21.

## 6. Summary and Conclusions

In this paper, a robust modeling strategy for reliable nonlinear FE analyses of RC structures was proposed. The modeling choices adopted for the reference FE model were validated through parametric studies and comparisons with other commonly used options; the effects of these choices on the numerical results were investigated. The contributions of this study are summarized as follows:

- Consistent approaches for deriving and defining the stress–strain relationships for concrete in tension and compression with consideration of the damage evolution and strain localization in the fracture zones of cracking and crushing concrete, respectively, were comprehensively described;
- Principles and recommendations for appropriately determining crack band width in structurally meshed concrete continuum to adjust the tensile stress–strain relationships were discussed and proved effective in avoiding the mesh sensitivity problem;
- An iterative implementation procedure was proposed to modify the concrete compressive stress–strain relationship in the post-peak regime according to the verified size of the crushing zone for considering the strain-localization effect;
- A simple and robust method was proposed for assigning the steel-concrete reinforcement interaction in ABAQUS using node-to-node connectors, and its accuracy was confirmed via a comparison with the commonly used method based on cohesive elements;
- Viscoplastic regularization using a properly defined viscosity parameter exhibited the capability to overcome the convergence difficulties encountered in the simulation of cracking or crushing concrete and thus significantly reduced the computational time;
- The proposed modeling strategy, as exemplified through the FE analyses of a reference RC beam, provided a reliable simulation of nonlinear responses including the development of cracks and resistance to concrete crushing. The ability to capture these effects on this simple case laid the foundation for the accurate modeling of CFRP debonding induced by intermediate flexural cracks in the strengthened beam, which provided an example of modeling RC structures with additional complexity.

Most of the recommendations presented in this paper for the nonlinear concrete model are general and applicable to FE analyses of other quasi-brittle material models with different FE software. The proposed modeling strategies can be directly used to model RC beam and frame structures with different dimensions, loading configuration, and boundary conditions. The current study also paves the way to FE analyses with higher degrees of complexity.

## Figures and Tables

**Table 1:** Material properties of concrete, steel rebars, CFRP plate, and cured epoxy adhesive.

Concrete C35/45		Steel Rebars B500C		CFRP Plate		Adhesive	
$E_{c}$	36.9 GPa	$E_{s}$	201 GPa	$E_{f}$	214 GPa	$E_{a}$	7.1 GPa
$f_{c}$	51.1 MPa	$f_{\mathrm{sy}}$	510 MPa	$\varepsilon_{\mathrm{fu}}$	12.7‰	$f_{a}$	34 MPa
$f_{\mathrm{ct}}$	3.6 MPa	$f_{\mathrm{su}}$	617 MPa	–	–	$\nu_{a}$	0.3
$\nu_{c}$	0.2	$\varepsilon_{\mathrm{su}}$	12.0%	–	–	–	–

**Table 2:** Plasticity parameters defined in the concrete damaged plasticity (CDP) model.

Categories	Plastic Flow Potential		Yield Surface		Viscosity Parameter
**Parameters**	φ	ϵ	$\sigma_{b0}/\sigma_{c0}$	$K_{c}$	μ
**Values**	35°	0.1	1.16	2/3	${1\;\times \;10}^{-6}$

**Table 3:** Three commonly used constitutive models for concrete in compression.

**Model Code**	$0<\varepsilon_{c}<\varepsilon_{c,\lim}$ $\sigma_{c}=f_{c}\left[k\frac{\varepsilon_{c}}{\varepsilon_{c1}}-{\left(\frac{\varepsilon_{c}}{\varepsilon_{c1}}\right)}^{2}\right]/\left[1+\left(k-2\right)\frac{\varepsilon_{c}}{\varepsilon_{c1}}\right]$ $\varepsilon_{c}>\varepsilon_{c,\lim}$ $\sigma_{c}=f_{c}{\left[\left(\frac{1}{e}\xi -\frac{2}{e^{2}}\right){\left(\frac{\varepsilon_{c}}{\varepsilon_{c1}}\right)}^{2}+\left(\frac{4}{e}-\xi \right)\frac{\varepsilon_{c}}{\varepsilon_{c1}}\right]}^{-1}$	where $k=E_{\mathrm{ci}}/E_{c1}$ $e=\varepsilon_{c,\lim}/\varepsilon_{c1}$ $\xi =4\frac{\left[e^{2}\left(k-2\right)\;+\;2e\;-\;k\right]}{{\left[e\left(k-2\right)\;+\;1\right]}^{2}}$ $\varepsilon_{c,\lim}=\varepsilon_{c,1}\left[\frac{1}{2}\left(\frac{k}{2}+1\right)+\sqrt{\frac{1}{4}{\left(\frac{k}{2}+1\right)}^{2}-\frac{1}{2}}\right]$
**Thorenfeldt**	$\sigma_{c}=\frac{E_{c}\varepsilon_{c}}{1+\left(\frac{E_{c}\varepsilon_{c1}}{f_{c}}-2\right)\left(\frac{\varepsilon_{c}}{\varepsilon_{c1}}\right)+{\left(\frac{\varepsilon_{c}}{\varepsilon_{c1}}\right)}^{2}}$	–
**Saenz**	$\sigma_{c}=f_{c}\frac{\varepsilon_{c}}{\varepsilon_{c1}}\left(\frac{n}{n-1+{\left(\frac{\varepsilon_{c}}{\varepsilon_{c1}}\right)}^{nk}}\right)$	$\mathrm{where}\;n=0.80+\frac{f_{c}}{17}$ $k=\{\begin{array}{cc} 1, & \;\;0<\varepsilon_{c}<\varepsilon_{c1}\\ 0.67+\frac{f_{c}}{62}, & \;\;\varepsilon_{c}>\varepsilon_{c1} \end{array}$

Note: $E_{\mathrm{ci}}=E_{c}$ represents the initial tangent modulus, $E_{c1}=f_{c}/\varepsilon_{c1}$ represents the secant modulus from the origin to the peak compressive stress, $\varepsilon_{c1}=1.60{\left(f_{c}/10\;\mathrm{MPa}\right)}^{0.25}/1000$ represents the strain corresponding to the peak compressive stress [48,53], and $\varepsilon_{c,\lim}$ represents the ultimate compressive strain of the concrete.

## Data Availability

The data that support the findings of this study are contained within the article.

## References

[1] Malm R., Holmgren J. (2008). Cracking in deep beams owing to shear loading. Part 2: Non-linear analysis. Mag. Concr. Res..

[2] Chen G.M., Teng J.G., Chen J.F. (2011). Finite-element modeling of intermediate crack debonding in FRP-plated RC beams. J. Compos. Constr..

[3] Grassl P., Johansson M., Leppänen J. (2018). On the numerical modelling of bond for the failure analysis of reinforced concrete. Eng. Fract. Mech..

[4] Hendriks M.A.N., de Boer A., Belletti B. (2017). Validation of the Guidelines for Nonlinear Finite Element Analysis of Concrete Structures—Part: Reinforced Beams.

[5] Schlune H., Plos M., Gylltoft K. (2012). Safety formats for non-linear analysis of concrete structures. Mag. Concr. Res..

[6] Jirásek M., Bažant Z.P. (2002). Inelastic Analysis of Structures.

[7] Chen G.M., Teng J.G., Chen J.F., Xiao Q.G. (2015). Finite element modeling of debonding failures in FRP-strengthened RC beams: A dynamic approach. Comput. Struct..

[8] Malm R. (2009). Predicting Shear Type Crack Initiation and Growth in Concrete with Non-Linear Finite Element Method.

[9] Camacho G.T., Ortiz M. (1996). Computational modelling of impact damage in brittle materials. Int. J. Solids Struct..

[10] May S., de Borst R., Vignollet J. (2016). Powell-Sabin B-splines for smeared and discrete approaches to fracture in quasi-brittle materials. Comput. Methods Appl. Mech. Eng..

[11] Wells G.N., Sluys L.J. (2001). A new method for modelling cohesive cracks using finite elements. Int. J. Numer. Methods Eng..

[12] Bažant Z.P., Oh B.H. (1983). Crack band theory for fracture of concrete. Mater. Struct..

[13] Rots J.G., Nauta P., Kuster G.M.A., Blaauwendraad J. (1985). Smeared crack approach and fracture localization in concrete. HERON.

[14] CEB-FIP (1993). CEB-FIP Model Code 1990: Design Code.

[15] Fib (2013). Fib Model Code for Concrete Structures 2010.

[16] American Concrete Institute (2014). ACI 318-14 Building Code Requirements for Structural Concrete and Commentary (Metric).

[17] Van Mier J.G.M. (1984). Strain-Softening of Concrete under Multiaxial Loading Conditions. Ph.D. Thesis.

[18] Bažant Z.P. (1989). Identification of strain-softening constitutive relation from uniaxial tests by series coupling model for localization. Cem. Concr. Res..

[19] Jansen D.C., Shah S.P. (1997). Effect of length on compressive strain softening of concrete. J. Eng. Mech..

[20] Zandi Hanjari K., Kettil P., Lundgren K. (2013). Modelling the structural behaviour of frost-damaged reinforced concrete structures. Struct. Infrastruct. Eng..

[21] Thorenfeldt E., Tomaszewicz A., Jensen J.J. Mechanical properties of high-strength concrete and applications in design. Proceedings of the Symposium on Utilization of High-Strength Concrete.

[22] Shu J., Fall D., Plos M., Zandi K., Lundgren K. (2015). Development of modelling strategies for two-way RC slabs. Eng. Struct..

[23] Dassault Systèmes (2014). Abaqus 6.14—Abaqus Analysis User’s Guide.

[24] Carloni C., D’Antino T., Sneed L.H., Pellegrino C. (2018). Three-dimensional numerical modeling of single-lap direct shear tests of frcm-concrete joints using a cohesive damaged contact approach. J. Compos. Constr..

[25] Ombres L., Verre S. (2020). Experimental and numerical investigation on the steel reinforced grout (SRG) composite-to-concrete bond. J. Compos. Sci..

[26] Dassault Systèmes (2014). Abaqus 6.14 Theory Manual.

[27] Malm R. (2016). Guideline for FE Analyses of Concrete Dams.

[28] Hanif M.U., Ibrahim Z., Jameel M., Ghaedi K., Aslam M. (2016). A new approach to estimate damage in concrete beams using non-linearity. Constr. Build. Mater..

[29] Alfarah B., López-Almansa F., Oller S. (2017). New methodology for calculating damage variables evolution in plastic damage model for RC structures. Eng. Struct..

[30] Kmiecik P., Kaminski M. (2011). Modelling of reinforced concrete structures and composite structures with concrete strength degradation taken into consideration. Arch. Civ. Mech. Eng..

[31] Labizadeh M., Hamidi R. (2017). Effect of stress path, size and shape on the optimum parameters of a brittle-ductile concrete model. Eng. Struct. Technol..

[32] Obaidat Y.T., Heyden S., Dahlblom O. (2010). The effect of CFRP and CFRP/concrete interface models when modelling retrofitted RC beams with FEM. Compos. Struct..

[33] Triantafillou T.C. (1998). Shear strengthening of reinforced concrete beams using epoxy-bonded FRP composites. ACI Struct. J..

[34] Hollaway L.C. (2010). A review of the present and future utilisation of FRP composites in the civil infrastructure with reference to their important in-service properties. Constr. Build. Mater..

[35] Cascardi A., Dell’Anna R., Micelli F., Lionetto F., Aiello M.A., Maffezzoli A. (2019). Reversible techniques for FRP-confinement of masonry columns. Constr. Build. Mater..

[36] CEN. EN 1992-1-1:2004 (2004). Eurocode 2: Design of Concrete Structures—Part 1-1: General Rules and Rules for Buildings.

[37] ASTM (2016). A615/A615M Standard Specification for Deformed and Plain Carbon-Steel Bars for Concrete Reinforcement.

[38] Yang J., Haghani R., Al-Emrani M. (2019). Innovative prestressing method for externally bonded CFRP laminates without mechanical anchorage. Eng. Struct..

[39] Heshmati M., Haghani R., Al-Emrani M. (2017). Durability of bonded FRP-to-steel joints: Effects of moisture, de-icing salt solution, temperature and FRP type. Compos. Part B Eng..

[40] Rabbat B.G., Russell H.G. (1985). Friction coefficient of steel on concrete or grout. J. Struct. Eng..

[41] Lubliner J., Oliver J., Oller S., Oñate E. (1989). A plastic-damage model for concrete. Int. J. Solids Struct..

[42] Lee J., Fenves G.L. (1998). Plastic-damage model for cyclic loading of concrete structures. J. Eng. Mech..

[43] Hordijk D.A. (1991). Local Approach to Fatigue of Concrete. Ph.D. Thesis.

[44] Pietruszczak S., Mróz Z. (1981). Finite element analysis of deformation of strain-softening materials. Int. J. Numer. Methods Eng..

[45] De Borst R. (1986). Non-Linear Analysis of Frictional Materials. Ph.D. Thesis.

[46] Rots J.G. (1988). Computational Modeling of Concrete Fracture. Ph.D. Thesis.

[47] Oliver J. (1989). A consistent characteristic length for smeared cracking models. Int. J. Numer. Methods Eng..

[48] Fib (2008). Bulletin 42: Constitutive Modelling of High Strength/High Performance Concrete.

[49] Tomaszewicz A. (1984). Betongens Arbeidsdiagram (Stress-Strain Relationship for Concrete), Report No. STF65 A84065.

[50] Collins M.P., Porasz A. (1989). Shear design for high strength concrete. CEB Bulletin D’information no. 193.

[51] Saenz L.P. (1964). Discussion of equation for the stress-strain curve of concrete-by Desayi, P. and Krishan, S. J. Am. Concr. Inst..

[52] Desayi P., Krishnan S. (1964). Equation for the stress-strain curve of concrete. J. Am. Concr. Inst..

[53] Popovics S. (1973). A numerical approach to the complete stress-strain curve of concrete. Cem. Concr. Res..

[54] Yao L.Z., Wu G. (2016). Nonlinear 2D finite-element modeling of RC beams strengthened with prestressed NSM CFRP reinforcement. J. Compos. Constr..

[55] Lu X.Z., Teng J.G., Ye L.P., Jiang J.J. (2005). Bond-slip models for FRP sheets/plates bonded to concrete. Eng. Struct..

[56] Jankowiak T., Lodygowski T. (2005). Identification of parameters of concrete damage plasticity constitutive model. Found. Civ. Environ. Eng..

[57] Esmaeeli E. (2015). Development of Hybrid Composite Plate (HCP) for the Strengthening and Repair of RC Structures. Ph.D. Thesis.

[58] Lee J., Fenves G.L. (1998). A plastic-damage concrete model for earthquake analysis of dams. Earthq. Eng. Struct. Dyn..

